# Prevalence of voice handicap among nurses in intensive care units due to occupational noise during pandemic

**DOI:** 10.3389/fpubh.2023.1250512

**Published:** 2023-09-01

**Authors:** Ziwei Song, Pyoung-Jik Lee, HeeJung Jung

**Affiliations:** ^1^Acoustics Research Unit, School of Architecture, University of Liverpool, Liverpool, United Kingdom; ^2^Department of Management and Entrepreneurship, Imperial College London, London, United Kingdom

**Keywords:** voice handicap, nurses, intensive care unit, occupational health, communication, pandemic (COVID-19), voice handicap index

## Abstract

**Background:**

Healthcare workers have been identified as being at risk of occupational voice disorders. Among them, nurses working in intensive care units (ICUs) are particularly vulnerable due to the risk factors that are associated with their exposure to high levels of noise. Thus, this study aimed to determine the prevalence of voice disorders among ICU nurses.

**Methods:**

A questionnaire was administered to 100 ICU nurses from four hospitals in China. The questionnaire assessed vocal-related symptoms, perceived voice handicap, frequently heard noise sources, and the quality of communications.

**Results:**

Results indicate that the most frequently reported voice symptoms were ‘voice tiredness’ and ‘voiceless’. Nurses working more than 50 h per week experienced voice symptoms more frequently than nurses working for 40–50 h per week. The median value of the perceived voice handicap score (VHI-30) was 23, indicating mild voice handicap, while 24% of the nurses reported severe voice handicap. Longer working hours and working at patient wards were significantly associated with higher VHI-30 scores. The nurses also reported that the quality of verbal communication with patients and colleagues and voice problems worsened during the COVID-19 pandemic.

**Conclusion:**

More than 20% of nurses reported severe voice handicap, however, voice handicap among ICU nurses did not appear universally to all nurses. Further research is necessary to identify the risk factors associated with voice disorders and the mechanism behind such heterogeneity among ICU nurses.

## 1. Introduction

Vocal problems are prevalent across various occupations, including teachers, singers, and acting professionals (1–3). Among them, teachers have been extensively studied, demonstrating a higher prevalence of voice disorders compared to the general population (4). Smith et al. (5) analysed 242 respondents from American teachers, revealing around 50% of them experienced hoarseness, followed by difficulties with high notes and a tired voice. Similarly, Munier and Kinsella (3) noted that primary school teachers commonly reported voice fatigue, dry throat and an inability to sing high notes as their predominant voice-related symptoms. Similar symptoms were reported among physical education teachers and head teachers in Iceland, including dry throat and vocal tiredness (6). Preciado and Infante (7) found an association between higher classroom noise levels and increased frequency of vocal disorders. Likewise, Servilha et al. (8) identified excessive classroom noise as a significant risk factor for the development of dysphonia and phonotrauma. Professional singers also face comparable challenges, frequently reporting hoarseness and voice fatigue (9). Pestana et al. (10) highlighted hoarse voice and tired voice as prevalent voice disorder symptoms among Fado singers.

Verdolini and Ramig (11) listed other occupational categories prone to voice disorders, such as lawyers, clergy and healthcare workers. They pointed out that more attention is required for healthcare workers and teachers who constitute the largest proportion among these categories. However, research on voice disorders among healthcare workers is limited and the findings are not consistent. Sala et al. (12) found that hospital nurses had a lower prevalence of voice disorders compared to teachers, although the noise exposure levels and frequency of voice use differed since the nurses were randomly selected from the hospital. Among nurses, the most common voice symptom was throat clearing, followed closely by voice tyres easily and sensation of a sore throat or globus. On the other hand, Heider et al. (13), in a recent questionnaire survey during the COVID-19 pandemic, reported that healthcare workers self-rated their voice handicap, indicating their being at risk of voice disorders (13). Similarly, Ribeiro et al. (14) observed that healthcare workers engaging in essential duties while wearing face masks were the ones most frequently beset by voice tiredness and voice impairment.

Noise levels in hospitals often exceed the recommendations set by the World Health Organisation (WHO) (15) of 35 dBA during the day and 30 dBA at night (16–18). Intensive care units (ICUs) face particularly high noise levels, with occasional peaks of 100 dBA due to alarm noises from medical equipment (17). Achieving the WHO’s recommended values is improbable without shutting down all medical equipment and ventilation are switched off. In facing elevated background noise, workers tend to increase their voice intensity, leading ICU nurses to speak louder to effectively communicate with patients and colleagues. For instance, Stringer et al. (19) highlighted the need for nurses to raise their voices significantly in operating theatres with excessive noise levels. Despite the potential risks associated with excessive noise in ICUs, its impact on nurses’ voices and investigations into voice disorders among ICU nurses have been largely overlooked. Instead, the nurses’ voice in the ICU has been considered a risk factor affecting patient’s sleep during night-time (20). Not only do individual nurses experience potential adverse impact, but organisations and hospitals also face significant disruption in their operation due to the dissatisfaction and subsequent turnover among their professional workers such as medical staffs (21). Therefore, it is necessary to understand how the nature of occupational environment affects occupational health among medical staff.

The present study aims to explore the voice problems experienced by ICU nurses. A questionnaire survey was conducted to rate the frequency of voice symptoms and assess the perceived voice handicap and changes in communication quality during the pandemic.

## 2. Materials and methods

### 2.1. Sampling

A total of 100 ICU nurse participants were recruited from four hospitals in Chongqing, China: 20 from Site A, 40 from Site B, 20 from Site C, and 20 from Site D. All the hospitals were located in close proximity to major traffic roads. They had different types of rooms in their ICUs, but all the rooms had an identical ceiling height of 2.8 m. Site A was built in 1937, and since then, no renovations have been conducted. ICU in Site A has three rooms with outdated equipment: one two-bedded room, one five-bedded room and one six-bedded room. Site B is the largest hospital in this research, built in 1943 and renovated in 1997. Its ICU has two one-bedded rooms, one two-bedded room, two six-bedded rooms and one 10-bedded room. Site C, built in 1985, has one 10-bedded room. Site D, built in 1943 and renovated in 2006, has one 18-bedded room and two one-bedded rooms. Hospital D is affiliated with the local University of Chongqing and has the most updated equipment. The socio-demographic and professional characteristics of the survey participants are presented in Table 1. The participants’ ages ranged from 25 to 38 years (mean: 34.6, SD: 3.2), with the majority being females. A significant proportion (74%) held the job title of a registered nurse and were responsible for providing direct care to patients in the ICU, working in rotating shifts. Approximately 14% of the participants were clinical nurses, responsible for ordering medical tests and developing treatment plans. Furthermore, 71% of the participants had worked in the ICUs for 5–10 years, with 22 participants having worked for more than 10 years. Most participants (74%) worked less than 50 h per week, and all individuals wore face masks for more than 8 h each day. The majority of beds in the four ICUs were dedicated to general surgery, cancer, and respiratory disease. Noise levels at each site, as reported by a recent study (16), are also listed. The study monitored noise levels at multiple patient wards and nursing stations over a 24-h period. The averaged noise levels across the multiple locations were computed and listed in the table in terms of average (*L*_Aeq_) and maximum (*L*_AFmax_) noise levels. The average noise levels from the sites exceeded the WHO’s recommended values, surpassing 55 dBA. Furthermore, the maximum noise levels were significantly high, exceeding 90 dBA. In particular, Site D exhibited a maximum noise level of 104.2 dBA.

**Table 1:** Characteristics of nurses who took part in the questionnaire surveys.

Personal characteristics		Site A (*N* = 20)	Site B (*N* = 40)	Site C (*N* = 20)	Site D (*N* = 20)	Total (*N* = 100)
Gender						
	Male	0	1	2	0	3
	Female	20	39	18	20	97
Age (years)						
	20–25	0	0	0	0	0
	25–30	5	13	3	3	24
	30–35	13	19	16	17	65
	>35	2	8	1	0	11
Job title						
	Nurse manager	1	1	1	1	4
	Clinical nurse	3	7	2	2	14
	Charge nurse	2	3	2	1	8
	Registered nurse	14	29	15	16	74
Working area						
	Nurse station	2	5	2	4	13
	Patient wards	18	35	18	16	87
Years of working						
	<1	0	0	0	0	0
	1–2	0	0	0	0	0
	2–5	3	1	2	1	7
	5–10	14	28	12	17	71
	>10	3	11	6	2	22
Hours of working per week						
	<40	0	0	0	0	0
	40–50	15	33	12	14	74
	50–60	5	5	8	4	22
	>60	0	2	0	2	4
Hours of wearing a face mask a day						
	1–4	0	0	0	0	0
	4–8	0	0	0	0	0
	8–12	20	40	20	20	100
Smoking						
	Yes	1	0	2	2	5
	No	19	40	18	18	95
Consumption of alcoholic beverage in the last 12 months						
	Yes	3	5	2	2	12
	No	17	35	18	18	88
Asthma						
	Yes	0	0	1	0	1
	No	20	40	19	20	99
Noise levels (dBA)						
	Average noise level^*^	54.6	56.8	55.2	55.8	
	Maximum noise level^**^	92.3	95.2	92.7	104.2	

* A-weighted equivalent noise level (*L*_Aeq_).

** A-weighted maximum fast time-weighted noise level (*L*_AFmax_).

### 2.2. Instrument

All participants were instructed to answer the questionnaire, consisting of three main parts: (1) basic information including demographic, (2) vocal problems in ICUs, and (3) noise sources and communication qualities. Firstly, participants were asked about the basic demographic backgrounds such as their gender and age, and other personal information such as smoking and alcohol consumption history. Additionally, they were asked about their health condition such as any past or current experience with asthma and respiratory allergies that would potentially affect the voice handicap (22). Secondly, participants were asked to rate their experience of vocal symptoms using a five-point scale (‘1’: never happened and ‘5’: always happens). The self-perception of voice handicaps was assessed using the Voice Handicap Index (VHI-30) developed by Jacobson et al. (23). The VHI questions consist of three components—i.e., ‘functional’, ‘physical’, and ‘emotional’. Each component includes 10 items, and each item is assessed on a five-point scale (0 = never, 4 = always). The total score from the 30 items is utilised to identify individuals with voice disorders, using a cut-off point of 30. The VHI-30 was translated into Chinese and districted to participants (24). Thirdly, frequency of noise sources heard in the ICUs were assessed using a five-point scale from ‘Do no hear at all’ to ‘Dominates completely’. Furthermore, participants were asked about their communication with patients and colleagues within the ICUs.

### 2.3. Ethics consideration

Participants were recruited for the study after obtaining approval from the research ethics committee of a large state university in the United Kingdom, as well as the ethics committees of four participating hospitals in China. The participants were provided with detailed information about the study procedures, and their consent to participate was obtained. They were assured that their participation would be kept confidential, and they had the freedom to withdraw from the study at any time.

### 2.4. Data analysis

Statistical analyses were conducted using SPSS-25 software (SPSS Inc., Chicago, IL, United States) and Minitab 20. The reliability of the VHI-30 scales was assessed using Cronbach’s test to ensure accurate understanding among participants. Differences in voice symptoms between nurses with varying levels of working experience and working hours were determined using the Mann–Whitney U-test. Correlation analysis was employed to assess the relationships between VHI-30 scores and measured noise levels. Differences in VHI-30 scores across the sites were evaluated using the Kruskal-Wallis test. The overall score of the VHI-30 questionnaire was dichotomised, using a cut-off of 30 points. Univariate and multivariate logistic regression models were constructed to evaluate the association between scores above the 30-point cut-off and independent variables such as sociodemographic and working characteristics. Odds ratios with 95% confidence intervals (CI) were calculated, and the goodness-of-fit of the multivariate logistic regression models was assessed using the Hosmer-Lemeshow test. In reporting results, *p* values less than 5% (*p* < 0.05) were considered statistically significant.

## 3. Results

Table 2 presents the frequency and types of voice symptoms reported by nurses across four ICUs. The percentage of participants who experienced these symptoms was calculated, along with the mean ratings. The percentages were computed based on those who selected ‘3 (quite often)’, ‘4 (very often)’ and ‘5 (always)’ on a five-point scale. The most frequent voice symptoms reported were ‘voice tiredness’ (32.0%), ‘voiceless’ (29.0%), ‘difficulty in being heard’ (28.0%) and ‘sore throat when speaking’ (25.0%). There was a variation in voice symptoms across hospitals. Overall, ICU nurses at Site A showed the lowest frequency of experiencing voice symptoms (mean = 12.0, SD = 5.4). In terms of frequency, Sites B and D (mean = 29.5, SD = 5.6 for Site B and mean = 22.5, SD = 7.2 for Site D) showed more frequent symptoms compared to the other sites (mean = 12.0, SD = 5.4 for Site A and mean = 19.5, SD = 7.6 for Site C). In particular, approximately 40% of participants at Site B reported experiencing voice tiredness and voiceless symptoms, while 17.5% of participants experienced those symptoms in Site A, 27.5% for Site C, and 32.5% for Site D.

**Table 2:** Percentage of those who experienced voice-related symptoms and mean of frequencies across the sites.

	Site A		Site B		Site C		Site D		Total	
	%	Mean	%	Mean	%	Mean	%	Mean	%	Mean
Hoarseness	10.0	1.5	22.5	2.1	10.0	1.9	20.0	2.0	17.0	1.9
Voice tiredness	20.0	1.9	37.5	2.5	30.0	2.3	35.0	2.2	32.0	2.2
Voiceless	15.0	1.6	37.5	2.3	25.0	2.2	30.0	2.0	29.0	2.1
Dryness in the throat	5.0	1.5	27.5	2.3	20.0	1.8	15.0	2.0	19.0	1.9
Sore throat when speaking	10.0	1.8	32.5	2.3	25.0	2.2	25.0	2.2	25.0	2.1
Aphonia	10.0	1.2	27.5	1.9	10.0	1.8	15.0	1.7	18.0	1.8
Clearing the throat	20.0	2.0	25.0	2.1	25.0	2.0	15.0	2.0	22.0	2.1
Difficulty in being heard	15.0	1.5	35.0	2.4	25.0	2.0	30.0	2.2	28.0	2.1
Persistent dry cough	5.0	1.4	25.0	2.0	15.0	1.9	20.0	2.2	18.0	1.9
Lump in the throat	10.0	1.6	25.0	1.8	10.0	1.9	20.0	1.9	18.0	1.8

The heterogeneity in voice symptoms among ICU nurse were investigated. The participants were categorised into two groups based on their working experiences and weekly working hours. Firstly, the nurses were divided into two groups: one group with 5–10 years of experience (*N* = 71) and the other group with more than 10 years of experience (*N* = 22). Secondly, the participants were classified into one group working for 40–50 h per week (*N* = 74) and another group working for more than 50 h per week (*N* = 22). The percentages of participants experiencing voice symptoms and the mean ratings of frequencies are listed in Supplementary Tables S1 and S2. Nurses with more than 10 years of experience reported experiencing ‘voice tiredness’ and ‘dryness in the throat’ more frequently than those with 5–10 years of working experience. Mann–Whitney U-test indicated that the differences in these voice symptoms between the groups are significant (*p* < 0.05 for both). However, nurses with 5–10 years of experience reported more frequent experiences of other symptoms than those with more than 10 years of experiences; but, their differences were not statistically significant. Overall, participants working more than 50 h per week reported experiencing vocal symptoms more frequently than those working less than 50 years. For instance, 43.3% of nurses working more than 50 h per week reported experiencing voice tiredness. Mann–Whitney U-test demonstrated that working hours had significant effects on several symptoms (‘voice tiredness’, ‘dryness in the throat’, ‘aphonia’ and ‘lump in the throat’ for *p* < 0.05 and ‘voiceless’ for *p* < 0.01).

The results of VHI-30 are presented in Table 3. While the Cronbach’s alpha value was 0.79 for the whole sites, indicating good internal consistency, the Sites C and D exhibited acceptable consistency, with the values of Cronbach’s alpha were 0.6 (25–27). The median values of the total VHI-30 score were below 30 for all the sites, suggesting minimal voice-related handicap. However, 24 participants obtained total VHI-30 scores higher than 30, indicating that approximately 24% of ICU nurses may have a voice disorder. Among the three subscales, the scores in the functional subscale were higher than those in the other subscales for all the sites. The results of Kruskal-Wallis test revealed that the VHI-30 total score at Site B was significantly higher than those at the other sites (*p* < 0.01). Correlation analysis also indicated a significant relationship between VHI-30 scores and the measured noise levels (r = 0.525, p < 0.01). This suggests that noisier environments lead to more severe perceived voice handicap among ICU nurses.

**Table 3:** Median values of the VHI-30 scores.

	Total (*N* = 100)	Site A (*N* = 20)	Site B (*N* = 40)	Site C (*N* = 20)	Site D (*N* = 20)
Total VHI-30	23	20	29	20	22.5
Functional subscale	8	6.5	10	8	8
Emotional subscale	7	6	8	7	7.5
Physical subscale	7.5	6.5	9.5	6.5	6
Cronbach’s Alpha	0.79	0.72	0.73	0.60	0.60

As shown in Table 4, the univariate logistic regression models revealed significant associations between several factors and the VHI-30 overall score among ICU nurses. Age, working area, working hours, smoking and alcohol consumption were found to be statistically significant. ICU nurses aged between 30 and 35 years obtained significantly higher scores (mean 1.28; 95% CI 0.58–1.97) compared to those aged between 25 and 30. Moreover, nurses working in patient wards displayed an overall score that was 0.19 (95% CI 0.03–0.35) higher than those working in the nurse station. Additionally, ICU nurses working for 50–60 h per week exhibited higher scores than those working for 40–50 h per week. Specifically, nurses working for more than 60 h per week had a score that was 3.82 (95% CI 0.09–7.55) higher than those working for 40–50 h per week. Furthermore, nurses who reported smoking or consuming alcoholic beverages within the last 12 months had higher overall scores compared to non-smokers and non-drinkers. Furthermore, in the multivariate logistic regression model, working area and working hours remained significantly associated with the VHI-30 overall score. Specifically, ICU nurses working in patient wards had a score that was 0.09 (95% CI 0.19–0.39) higher than those working in the nurse station. Moreover, nurses who worked for more than 60 h per week displayed a score that was 1.13 (95% CI 0.05–3.24) higher than those working for 40–50 h per week.

**Table 4:** Univariate and multivariate logistic regression analyses for VHI-30 scores over the cut-off (dependent variable) and the independent variables of sociodemographic and working characteristics (*n* = 100).

	VHI > 30 points n (%)	Univariate models, OR (95% CI)	*p* value	Multivariate model, OR (95% CI)	*p* value
Age (years)					
25–30	9/24 (37.5)	Reference		Reference	
30–35	14/65 (4.6)	**1.28 (0.58–1.97)**	**0.041**	2.80 (0.21–3.73)	0.435
>35	1/11 (18.2)	1.69 (0.28–3.10)	0.409	0.71 (0.57–8.64)	0.782
Job title					
Nurse manager	1/4 (25.0)	Reference			
Clinical nurse	3/14 (21.4)	1.62 (0.32–11.97)	0.976		
Charge nurse	1/8 (12.5)	1.47 (0.16–13.96)	0.737		
Registered nurse	19/74 (25.7)	1.42 (0.28–6.01)	0.512		
Working area					
Nurse station	15/87 (17.2)	Reference		Reference	
Patient wards	9/13 (69.2)	**0.19 (0.03–0.35)**	**<0.001**	**0.19 (0.28–1.25)**	**0.024**
Years of working					
2–5	2/7 (28.5)	Reference			
5–10	16/71 (22.5)	1.30 (0.20–2.40)	0.754		
>10	6/22 (27.3)	2.53 (0.35–4.70)	0.881		
Hours of working per week					
40–50	16/74 (21.6)	Reference		Reference	
50–60	6/22 (22.7)	**1.03 (0.14–1.92)**	**0.026**	0.13 (0.10–5.24)	0.570
>60	2/4 (50.0)	**3.82 (0.09–7.55)**	**0.022**	**1.81 (0.64–3.38)**	**0.042**
Smoking					
No	22/95 (23.2)	Reference		Reference	
Yes	2/5 (40.0)	**1.83 (0.11–8.91)**	**<0.001**	0.64 (0.06–6.62)	0.711
Consumption of alcoholic beverage in the last 12 months					
No	18/88 (21.6)	Reference		Reference	
Yes	6/12 (50.0)	**3.72 (0.12–13.49)**	**<0.001**	0.14 (0.02–0.87)	0.035

^*^Variables significantly associated with VHI-30 overall score in bold. ^**^The multivariate model only included variables that were associated with the VHI-30 overall score in the univariate models. ^***^The Hosmer-Lemeshow goodness-of-fit test was non-significant (*p* = 0.22).

**Figure 1:**
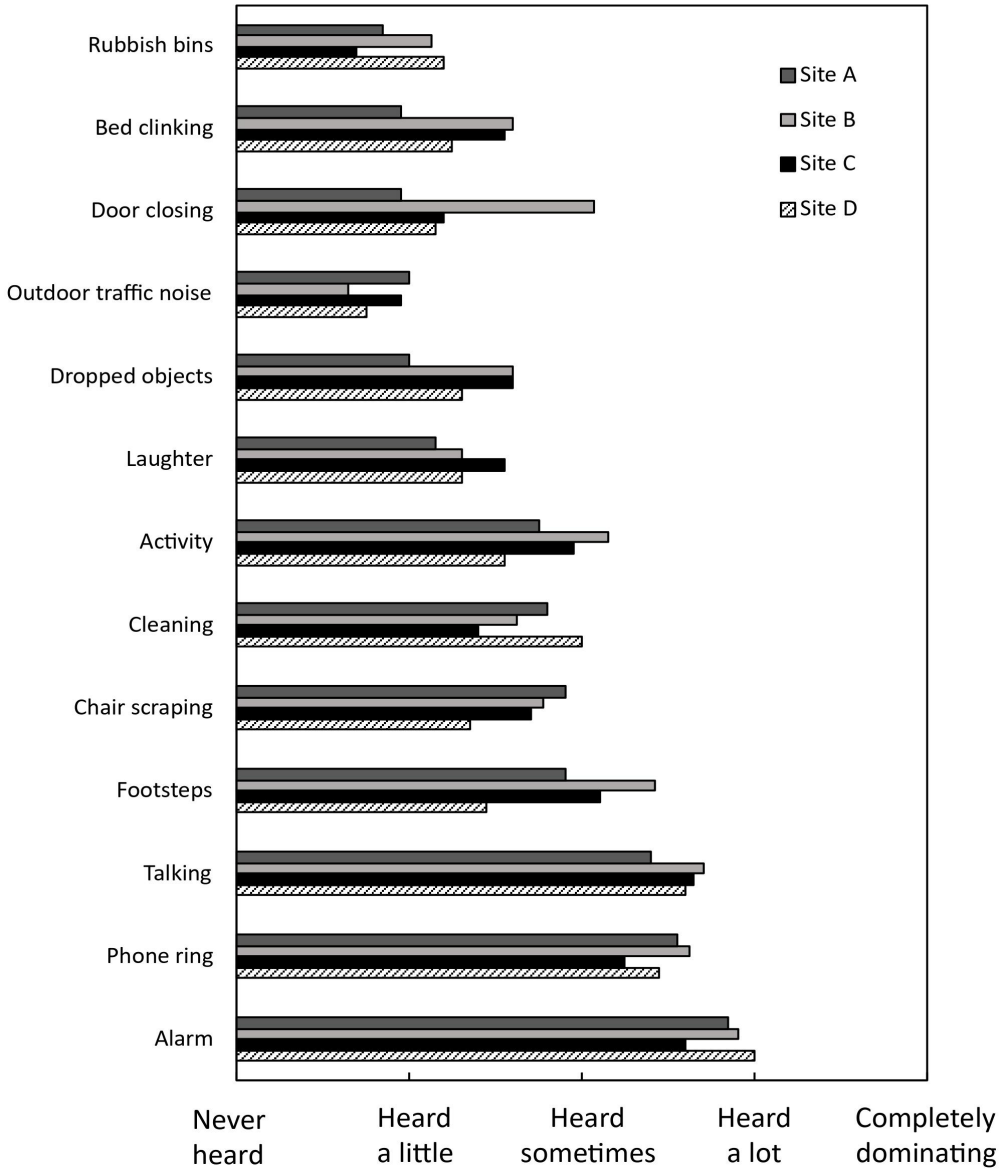
Mean ratings of respondents’ perceived occurrence of different sounds, indicating the frequency of heard noise sources across the sites.

Figure 1 shows the extent to which noise sources are frequently heard in the ICU for each site. The most commonly heard noise source was alarms from medical equipment, followed by talking, phone rings, footsteps and general activities like typing on a keyboard and closing drawers. The dominant noise sources were consistent across the sites, but there were some variations observed. For example, Site B had higher frequencies of door closing and footsteps compared to the other sites.

**Figure 2:**
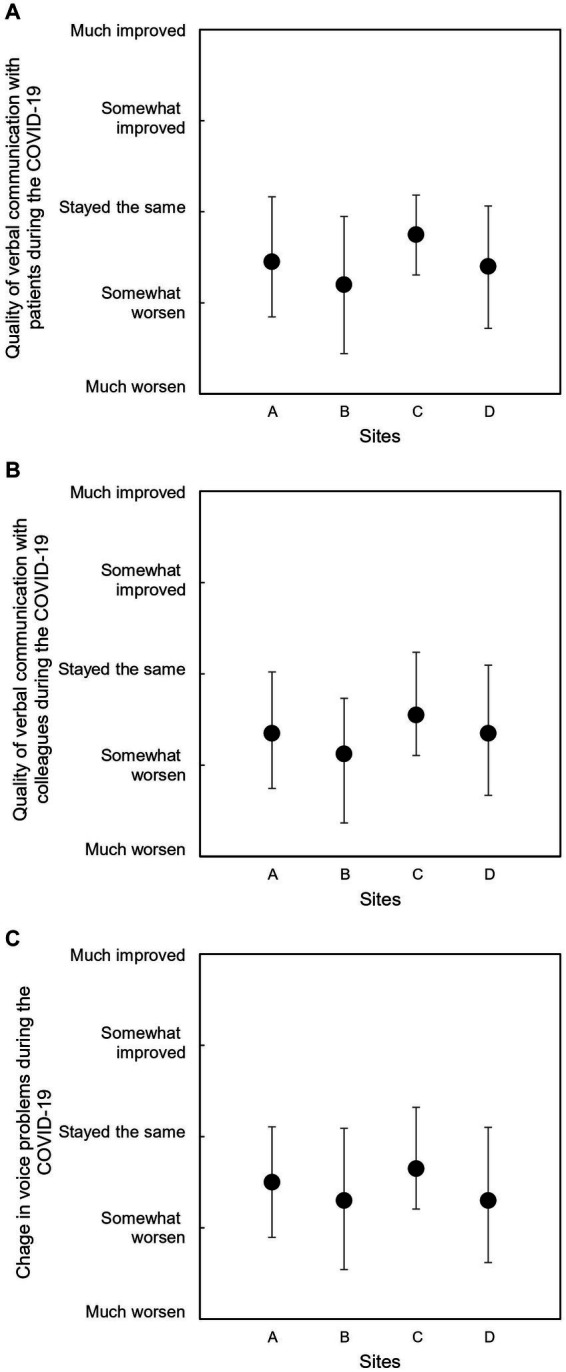
Mean ratings of communication quality and voice problems during the COVID-19 pandemic: **(A)** verbal communication with patients, **(B)** verbal communication with colleagues, and **(C)** voice problems. Error bars indicate standard deviations.

The changes in the quality of verbal communications with patients and colleagues and the severity of voice problems during the COVID-19 pandemic are investigated using a five-point scale (1 = much worsen and 5 = much improved), presented in Figure 2. All the responses were below 3 (indicating ‘stayed the same’), suggesting that communication quality and voice problems that ICU nurses experienced worsened during the pandemic. Site B displayed the lowest scores for all questions, while Site C had the highest scores.

**Figure 3:**
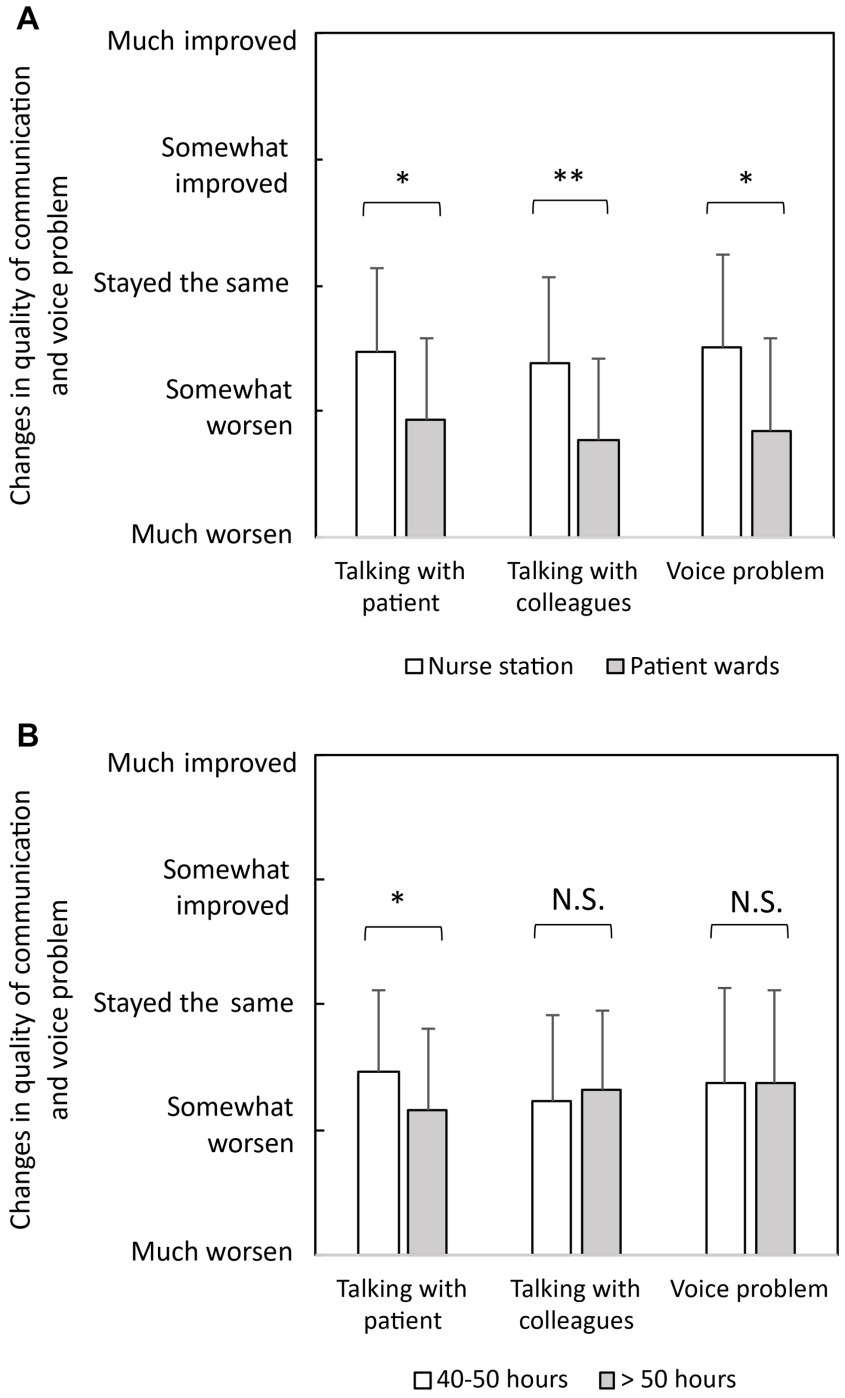
Comparisons of communication quality and voice problems during the COVID-19 pandemic for different groups: **(A)** nurses with different working hours per week and **(B)** nurses with different working locations. Error bars indicate standard deviations. ^*^p < 0.05, ^**^p < 0.01.

Figure 3 illustrates the comparisons among nurses with different working hours and working locations. As depicted in Figure 3A, nurses working more than 50 h per week exhibited significantly poorer quality of communication with patients during the pandemic compared to those working 40–50 h per week. However, no significant differences were observed between the groups in terms of communication with colleagues and voice problems (*p* > 0.05). Additionally, as depicted in Figure 3B, nurses working in patient wards reported significantly worse experiences in communicating with patients, colleagues and exhibited more voice problems compared to nurses working at nurse stations. These findings suggest that ICU nurses who had more time and opportunities for verbal communication experienced lower communication quality and more voice problems during the pandemic.

## 4. Discussion

This study showed that the handicap associated with voice disorders in ICU nurses was mild (<30) based on the VHI-30 scores. Similar findings have been reported in several studies involving voice-related professionals, where the mean VHI scores were also below the cut-off points (30 for VHI-30 and 11 for VHI-10). For example, studies on teachers with voice disorders (28–30), student teachers (31), voice professionals (32–34) and Imams (35) reported mean VHI-30 scores below 30. Furthermore, Heider et al. (13) found that only 26.2% of healthcare workers exhibited moderate or severe symptoms based on the VHI-10. Similarly, the percentage of people who scored above the abnormal cut-off point was 8.2% among military service members working in noisy environments (36). However, there are also studies that reported moderate to severe disability in teachers based on the VHI-30 (37). Similarly, Lyberg-Åhlander et al. (38) found a higher prevalence of voice disorders among teaching-related professionals compared to health professionals. Therefore, further research is needed to assess voice disorders in ICU nurses in diverse environments and compare them to other occupations.

Mild voice handicap, as found in this study, does not imply that ICU nurses are entirely free from the risk of voice disorders. The evidence from four hospitals showed that 24% of ICU nurses had VHI-30 scores exceeding 30, suggesting the possibility of a moderate voice disorder. Furthermore, the questionnaire survey identified several voice problems among ICU nurses. Approximately 30% of nurses reported experiencing ‘voice tiredness’, ‘voiceless’, and ‘difficulty in being heard’. These symptoms highlight the need for increased attention to protect ICU nurses from such issues. Prior to the pandemic, Sala et al. (12) conducted a questionnaire survey with nurses and found that only 11% reported experiencing voice tiredness. The disparity in results may be attributed to the current study being conducted during the pandemic, which necessitated the use of face masks. Similarly, Aliabadi et al. (39) reported varying decreases in speech intelligibility among nurses using different types of personal protective equipment (PPE), such as surgical masks and face shields.

Among the four sites, Site B exhibited a higher median VHI-30 score compared to the other sites. Similarly, nurses from Site B reported experiencing voice-related symptoms such as ‘voice tiredness’ and ‘voiceless’ more frequently than their counterparts at other sites. These differences can be attributed to variations in working conditions and acoustic environments across the sites. Site B, with 26 patient beds, has a larger number compared to the other sites (Site A: 14, Site C: 13 and Site D: 20), potentially resulting in more sources of noise. As listed in Table 1, average noise levels over a 24-h period were higher at Site B compared to the other sites. The median values of the sound exposure levels and maximum SPL of talking/voice at Site B were approximately 80 and 70 dBA, respectively (16). These findings suggest that noisier environments contribute to an increased occurrence of voice-related symptoms and voice handicap among ICU nurses.

The number of working hours was identified as an independent variable significantly associated with higher VHI-30 scores, indicating a higher perception of vocal handicap among ICU nurses. This finding supports the hypothesis of occupational health risks related to vocal disorders in ICU nurses, aligning with previous studies (16, 40). Heider et al. (13) reported that both the number of working hours and the duration of mask usage per day were risk factors for perceived vocal handicap in healthcare workers. Similarly, a higher number of voice usage hours per week was found to be a risk factor for voice handicap among student teachers (40). While ICU nurses tend to speak less frequently during working hours compared to teachers (41), the excessive noise in the ICU environment may lead to increased vocal effort and potential voice disorders. Given the constraint of reducing working hours for ICU nurses, it becomes crucial to improve the working environment by reducing noise levels in ICUs.

In this study, most nurses in ICUs wore surgical masks during their shifts, necessitating the assessment of speech transmission characteristics in a laboratory setting. The surgical face masks were placed on a Head & Torso Simulator (Type 4128C, B&K), and measurements of Speech Transmission Index (STI) and sound pressure level (SPL) were conducted at a distance of 1 m from the simulator. The study found minimal changes in STI values, ranging from 2.2 to 3.5%. Further details can be found in Supplementary Table S3. These results are consistent with previous research, which has reported a 3–4% decrease in STI for surgical masks (42) and a 3–6% decrease for disposable masks (43). Despite the minimal effect on STI values, the results of the questionnaire survey revealed that communication and voice problems worsened during the pandemic due to the use of PPE such as face masks. This finding aligns with other studies (14, 44) that have reported negative effects of PPE on verbal communication. Hampton et al. (44) found a significant impact of wearing PPE on speech discrimination in healthcare environments, while the use of face masks led to increased self-reported vocal discomfort and communication difficulties (14). Furthermore, the deterioration of communication in ICUs can be attributed to the lack of visual cues, including facial gestures and lip movements, which play a crucial role in understanding speech, particularly when speech quality is degraded.

There are a couple of limitations to consider in this study. First, this study only recruited ICU nurses and thus it is unclear the findings around the voice handicap is applicable to other similar occupations. Ideally, this study can be extended to different occupational categories of healthcare professionals (e.g., doctors and caregivers), to different demographics (e.g., status and language), to different department (e.g., emergency room) or to different countries. Secondly, this study focused on measuring noise levels in ICUs, which were found to be quite similar. However, it is important to note that individual nurses may have different levels of noise exposure. Therefore, it is recommended to conduct individual noise monitoring for nurses to accurately assess their personal noise exposure levels. Third, this study was interested in the finely defined symptom of voice disorders. However, nurses may have other adverse experiences due to the noise such as reduced well-being, job satisfaction and turnover. Finally, this study is not able to identify the root causes behind the heterogeneity in experiencing voice problem across nurse groups. Understanding the underlying causes is crucial, as it holds the potential to provide avenues for mitigating occupational health risks and thereby enhancing overall occupational quality. This may be resolved by conducting the follow-up survey or investigating the contents of communication. Therefore, further studies are necessary to examine ICU nurses’ experiences and perceptions in a more comprehensive manner. To do so, a larger number of nurses could be recruited in the future as the current study has only a small size of sample.

## 5. Conclusion

The present study revealed that ICU nurses frequently experienced voice-related symptoms, and longer working hours amplified these experiences. Furthermore, the study confirmed the prevalence of voice handicap among ICU nurses using the VHI-30 assessment. Specifically, the study identified working area and weekly workload as risk factors for perceived voice handicap. These findings emphasise the potential impact of prolonged and intense work schedules, particularly in noisy environments like ICUs, on the vocal health of nurses. The study’s results suggest the need for interventions and support systems to address and mitigate the adverse effects of noise on the vocal well-being of healthcare professionals. Moreover, the study’s findings highlight the importance of considering the unique challenges faced by nurses during the COVID-19 pandemic, which have further exacerbated their vocal difficulties and communication issues due to the wearing of PPEs.

## Data availability statement

The original contributions presented in the study are included in the article/Supplementary material, further inquiries can be directed to the corresponding author.

## Ethics statement

The studies involving humans were approved by Central ethics committee of the University of Liverpool. The studies were conducted in accordance with the local legislation and institutional requirements. The participants provided their written informed consent to participate in this study.

## Author contributions

PJL designed the study. ZS collected and analysed the data and drafted the first version of the manuscript. PJL and HJ critically revised the manuscript. All authors contributed to the article and approved the submitted version.

## Conflict of interest

The authors declare that the research was conducted in the absence of any commercial or financial relationships that could be construed as a potential conflict of interest.

## Publisher’s note

All claims expressed in this article are solely those of the authors and do not necessarily represent those of their affiliated organizations, or those of the publisher, the editors and the reviewers. Any product that may be evaluated in this article, or claim that may be made by its manufacturer, is not guaranteed or endorsed by the publisher.
