# Supplementary material for: Prevalence of voice handicap among nurses in intensive care units due to occupational noise during pandemic
Source: Front Public Health. 2023 Sep 1;11:1250512. doi: 10.3389/fpubh.2023.1250512 (PMC10505665; doi:10.3389/fpubh.2023.1250512)
Supplement: Supplementary file 1 [file Table_1.DOCX]

Supplementary Material

Prevalence of voice handicap among nurses in intensive care units due to occupational noise

**Ziwei Song, Pyoung Jik Lee^*^, HeeJung Jung**

*** Correspondence:** Pyoung-Jik Lee, p.j.lee@liverpool.ac.uk

**Supplementary Table S1.** Percentage of those who experienced voice-related symptoms and mean of frequencies for two groups with different working years. Asterisks indicate Mann-Whitney U-test results (*p<0.05).

|  | 5-10 years (*N*=71) | | >10 years (*N*=22) | |
| --- | --- | --- | --- | --- |
|  | % | Mean | % | Mean |
| Hoarseness | 21.1 | 2.0 | 4.0 | 1.8 |
| Voice tiredness* | 33.8 | 2.2 | 36.0 | 2.2 |
| Voiceless | 31.0 | 2.2 | 24.0 | 2.0 |
| Dryness in the throat* | 19.7 | 2.0 | 28.0 | 2.0 |
| Sore throat when speaking | 28.2 | 2.1 | 16.0 | 2.0 |
| Aphonia | 18.3 | 1.8 | 12.0 | 1.6 |
| Clearing the throat | 29.6 | 2.1 | 12.0 | 2.0 |
| Difficulty in being heard | 32.4 | 2.2 | 8.0 | 1.9 |
| Persistent dry cough | 18.3 | 1.9 | 16.0 | 1.9 |
| Lump in the throat | 23.9 | 2.0 | 16.0 | 1.8 |

**Supplementary Table S2.** Percentage of those who experienced voice-related symptoms and mean of frequencies for two groups with different working hours per week. Asterisks indicate Mann-Whitney U-test results (*p<0.05 and **p<0.001).

|  | 40-50 hours (*N*=74) | | >50 hours (*N*=22) | |
| --- | --- | --- | --- | --- |
|  | % | Mean | % | Mean |
| Hoarseness | 19.4 | 1.9 | 16.7 | 1.8 |
| Voice tiredness* | 27.4 | 2.1 | 43.3 | 2.4 |
| Voiceless** | 27.4 | 2.1 | 36.7 | 2.2 |
| Dryness in the throat* | 17.7 | 1.9 | 23.3 | 2.0 |
| Sore throat when speaking | 27.4 | 2.1 | 23.3 | 2.1 |
| Aphonia* | 16.1 | 1.7 | 20.0 | 1.9 |
| Clearing the throat | 25.8 | 2.1 | 16.7 | 1.9 |
| Difficulty in being heard* | 33.9 | 2.1 | 20.0 | 2.1 |
| Persistent dry cough | 27.4 | 2.0 | 26.7 | 1.8 |
| Lump in the throat* | 17.7 | 1.8 | 23.3 | 2.0 |

**Supplementary Table S3.** Sound pressure level (SPL) and Speech Transmission Index (STI) measurements at 1 m and 2 m away from the sound source across four surgical masks.

| Face mask | SPL | | STI | |
| --- | --- | --- | --- | --- |
|  | 1 m | 2 m | 1 m | 2 m |
| None | 68.4 | 65.3 | 0.91 | 0.87 |
| Surgical mask Type A-1 | 68.0 | 65.1 | 0.89 | 0.85 |
| Surgical mask Type A-2 | 68.0 | 64.9 | 0.90 | 0.84 |
| Surgical mask Type B-1 | 68.0 | 64.9 | 0.89 | 0.84 |
| Surgical mask Type B-2 | 67.9 | 65.0 | 0.88 | 0.84 |
